# Organ Restoration With Normothermic Machine Perfusion and Immune Reaction

**DOI:** 10.3389/fimmu.2020.565616

**Published:** 2020-10-19

**Authors:** Alessandro Parente, Daniel-Clement Osei-Bordom, Vincenzo Ronca, M. Thamara P. R. Perera, Darius Mirza

**Affiliations:** ^1^Liver Unit, Queen Elizabeth Hospital, University Hospitals Birmingham NHS Foundation Trust, Birmingham, United Kingdom; ^2^Centre for Liver and Gastrointestinal Research, Institute of Immunology and Immunotherapy, University of Birmingham, Birmingham, United Kingdom; ^3^National Institute for Health Research Birmingham Liver Biomedical Research Centre, University Hospitals Birmingham National Health Service Foundation Trust, Birmingham, United Kingdom; ^4^Division of Gastroenterology and Centre for Autoimmune Liver Diseases, Department of Medicine and Surgery, University of Milan Bicocca, Milan, Italy

**Keywords:** normothermic machine liver perfusion, immune activation, hepatic microenvironment, graft survival, liver transplantation

## Abstract

Liver transplantation is the only recognized effective treatment for end-stage liver disease. However, organ shortages have become the main challenge for patients and physicians within the transplant community. Waiting list mortality remains an issue with around 10% of patients dying whilst waiting for an available organ. The post-transplantation period is also associated with an adverse complication rate for these specific cohorts of high-risk patients, particularly regarding patient and graft survival. Ischaemia reperfusion injury (IRI) has been highlighted as the mechanism of injury that increases parenchymal damage, which eventually lead to significant graft dysfunction and other poor outcome indicators. The consequences of IRI in clinical practice such as reperfusion syndrome, primary non-function of graft, allograft dysfunction, ischaemic biliary damage and early biliary complications can be life-threatening. IRI dictates the development of a significant inflammatory response that drives the pathway to eventual cell death. The main mechanisms of IRI are mitochondrial damage due to low oxygen tension within the hepatic micro-environment and severe adenosine triphosphate (ATP) depletion during the ischaemic period. After the restoration of normal blood flow, this damage is further enhanced by reoxygenation as the mitochondria respond to reperfusion by releasing reactive oxygen species (ROS), which in turn activate Kupffer cells within the hepatic micro-environment, leading to a pro-inflammatory response and eventual parenchymal cell apoptosis and associated tissue degradation. Machine perfusion (MP) is one emergent strategy considered to be one of the most important advances in organ preservation, restoration and transplantation. Indeed, MP has the potential to rescue frequently discarded organs and has been shown to limit the extent of IRI, leading to suppression of the deleterious pro-inflammatory response. This immunomodulation reduces the prevalence of allograft rejection, the use of immunosuppression therapy and minimizes post-transplant complications. This review aims to update the current knowledge of MP with a focus on normothermic machine liver perfusion (NMLP) and its potential role in immune response pathways.

## Introduction

Liver transplantation (LT) is the only effective and definitive therapy for end stage liver disease. However, the increasing disparity between the number of organ donors and the number of wait-listed patients has become a constant challenge for the transplant community worldwide, with an overall waiting list mortality between 15 and 20% in the UK (1).

Based on these concerns, sub-optimal grafts, which are those from “Extended Criteria Donors” (ECDs), have been used for LT to ameliorate the difference between organ demand and availability. There are limited guidelines on the use of these grafts from ECDs, hence the numerous clinical trials being carried out to review the viability and functionality they may possess. ECDs are considered to be: (1) older aged (≥60 years old) brain death donors (DBD), (2) donors (aged 50–59 years old) with underlying medical co-morbidities(two of either stroke, hypertension, or serum creatinine >1.5 mg/dL), (3) donors with high-grade steatosis, and (4) cardiac death donors (DCD) or donors whose liver is subjected to prolonged cold ischaemia time (CIT). Historically, transplanted grafts from ECDs had poorer graft outcomes including primary non-function (PNF), early allograft dysfunction (EAD) and ischaemic cholangiopathy (2). MP protocols from clinical trials such as Dual Hypothermic Oxygenated Perfusion of DCD Liver Grafts (DHOPE) and Consortium on Organ Preservation in Europe (COPE) project have developed diverse methods to manipulate the immune response and restore marginal organs to a state where they are deemed transplantable. In this setting, the overall donor pool could be expanded, which could lighten the burden on the transplantation list and reduce waitlist mortality. This review will look at normothermic machine perfusion (NMP) and its effect on the immune reaction upon on the graft during the transplantation phase.

## The Rationale of Organ Preservation by Machine Perfusion

Static cold storage (SCS) has been the standard preservation method for liver grafts pre-transplantation. The basis of this preservation technique is inducing hypothermic conditions to reduce cellular activity, theoretically reducing the consumption of mitochondrial ATP substrate stores, increasing anaerobic metabolism and leading to an increase in lactate production within the parenchymal cells. Despite the fact that the liver microenvironment is ordinarily hypoxic, a further hypoxic insult is detrimental to hepatocytes. At low temperatures cells sustain a basal activity that, in a hypoxic environment, induces ATP depletion and lactic acid accumulation. Decreasing levels of ATP induce cellular lysis via the loss of the normal electrolyte cell membrane gradients, which triggers the activation of proteolytic enzymes (3). Mitochondrial damage develops due to lack of available metabolic substrates namely oxygen and ATP. This is the metabolic pathway and underlying cause of ischaemia reperfusion injury (IRI) (4). The period of oxygen deprivation, and then the restoration of normal oxygenated blood flow to the liver propagates the IRI cascade. After reperfusion the mitochondria react by releasing all the toxins generated and accumulated during the ischaemic period, which encompasses the time from donor graft vessel clamp time to cold ischaemia time. These toxins include the reactive oxygen species (ROS) which activate pro-inflammatory cascades and, eventually, cell apoptotic pathways (5–7).

Within the liver allograft, the oxygen deprivation that provokes the release of damage associated molecular patterns (DAMPs), such as HMGB-1, which promote the inflammatory response by activating Antigen Presenting Cells (APCs), like dendritic and resident immune cell subsets, Kupffer cells. However, ECD livers have a lower tolerance of hypoxia, leading to a more severe IRI and more serious clinical consequences, such as reperfusion syndrome, allograft dysfunction, ischaemic biliary damage and early biliary complications. There is currently an array of new preservation methods with varied perfusate fluid types and machine perfusion methods in normothermic machine perfusion. Nonetheless, hypothermic machine perfusion (HMP) and subnormothermic machine perfusion (SNMP) play a dynamic role in mediating the immune response in a number of visceral organ transplantations including the kidneys, liver, pancreas, intestine, heart and lung (8).

Machine perfusion (MP) has been key in preserving Donors after Circulatory Death (DCD) allografts. MP has shown promise in utilizing DCD kidneys for transplantation, with the MP-preserved renal allografts performing better when compared with simple cold storage (9–11). Taylor et al. highlighted the significance of this result, with the aim to widen the renal donor pool without fear of transplanting a graft susceptible to numerous post-operative complications and adverse long-term outcomes. There are some complications that occur more frequently in the early post-transplant phase in the MP DCD kidney group, including significantly higher incidences of early delayed graft function and primary non-function cases when compared with matched MP DBD donor grafts. However, on evaluation of long-term outcomes, renal function in the MP DCD kidney group recovers within 5–6 months post-transplantation, and both DCD and DBD donor kidneys have equal serum creatinine levels at this timepoint (12). Studies including Weissenbacher et al. have demonstrated that prolonged perfusion whilst recirculating urine in with the perfusate in order to maintain the composition of the perfusate fluid has beneficial effects on the graft and ensures stability of the perfusate (13).

NMP in liver (NMLP) and renal grafts reduce peak creatinine, improve graft survival, and allow organ recovery after periods of cold ischaemia. The energy requirements that the graft demands whilst being on NMP means that there is a requirement for an oxygen carrier, oxygen, and nutrient supplementation within the perfusion circuit to support physiological metabolic rates. By doing so research groups have reduced the effect of reperfusion tissue injury and subsequent graft dysfunction (14). Hypothermic Machine Perfusion (HMP) has clearly been shown to be advantageous in the preservation of both DBD and DCD allografts of the liver, kidneys, and pancreas. HMP in renal allografts lowers the incidence of poor clinical parameters in sub-optimal grafts post-renal transplantation (15, 16). HMP use has increased due to decreased Delayed Graft Function (DGF) occurrence for ECD, DCD, and DBD donor kidneys (17). With ECD, DCD, and DBD grafts, HMP allows an extensive review and assessment of the renal organ prior to transplantation, according to the “Predictive Score Model for Delayed Graft Function Based on Hypothermic Machine Perfusion Variables in Kidney Transplantation (18).”

There are similar studies of hypothermic machine perfusion of the pancreases, that demonstrated reduced graft oedema and reduced islet and acinar damage (19, 20). One study using 20 human pancreases in a hypothermic pulsatile perfusion of marginal human pancreas-duodenum organs for 24 h showed that this strategy was feasible with no deleterious parenchymal effect (21). Further observations highlighted that the ideal parameters for hypothermic continuous perfusion of pancreases has been difficult to ascertain. Branchereau et al. demonstrated that whilst using a hypothermic oxygenated continuous circuit at 25 mmHg for 24 h, none of the pancreas grafts used in the study showed signs of, or produced markers of, cellular injury, oedema, or necrosis. The tissue level of adenosine triphosphate (ATP) is a long-established marker of graft viability and functionality and is universal for all solid organ transplants (22). ATP is also a marker of the viability of machine-perfusion treated grafts (23). The Branchereau study highlighted that the tissue-biopsies of the pancreases in the HMP-group had a higher concentration of ATP levels when compared to the control group (21, 24). The maintenance of low pulsatile perfusion pressures in turn prevents endothelial damage and increased vasculature thrombosis (24, 25). We see that the low pressures and the pulsatile action may activate endothelial protective genes, including Kruppel-like factor 2, which is overexpressed by the endothelium during perfusion and has an antithrombotic action and promotes effective local microcirculation (26).

Huang et al. (27) used sub-normothermic machine liver perfusion (SNMLP) in whole and split liver NMLP. The study highlighted that ATP generation during perfusion is a potential viability marker that may be predictive of allograft function after transplantation. The ATP:ADP and ATP:AMP ratios increased similarly between split lobes and whole grafts within the first 2 h and decreased slightly by the 3rd hour among both the whole liver and split liver groups in this study. The increase of the energy ratios is an important marker for good graft function. No significant differences were seen when comparing left and right lobes for the energy ratios during the split machine perfusion model. This highlights the potential in the split technique, as we can hypothesize that both lobes will behave metabolically and physiologically in the same way (28). In essence, if each lobe has good function, we are therefore able to further increase the donor pool by utilizing what previously was one suboptimal liver allograft into two viable transplantable allografts (27, 29, 30).

With improving viability of grafts on MP, research groups often seek to use quantitative analysis of DAMPs (HMGB-1, cfDNA, etc.) and PAMPs (Pathogen-Associated Molecular Patterns) in tissue and perfusate as markers of graft restoration and the dampening of intraparenchymal immune reactions highlighted in various SNMP and HMP studies, such as the Huang and Porte groups, respectively. For extended perfusion and to improve graft function in most visceral allografts using HMP, the circuit requires oxygenation. Transforming the HMP into a hypothermic oxygenated perfusion further attenuates the immune reaction in liver donor grafts during extended MP restoration (31). Dual hypothermic oxygenated machine perfusion (DHOPE), a new clinical preservation technique developed by the Porte group, attempts to preserve donor liver grafts of poorer quality for longer periods of time (24 h). This facilitates transplantation by expanding the donor pool to include both marginal and sub-marginal human and porcine livers. The study highlighted that extended end-ischaemic DHOPE enabled successful preservation of porcine and discarded human liver grafts for up to 24 h (with required perfusion temperatures to be remain between 8 and 10°C) (31). Specific markers and cytokines were measured to compare and quantify hepatocellular injury and the production of deleterious pro-inflammatory cytokines. Alanine aminotransferase (ALT), HMGB-1, and cell-free DNA (cfDNA) (32) are markers used to detect and quantify nuclear subcellular injury and cell damage with necrosis. Tumor necrosis factor alpha (TNF-α) and interleukin 6 (IL-6) were markers of initiation and development of a proinflammatory response (33), whilst malondialdehyde (MDA) was used to detect lipid peroxidation seen in oxidative stress. MDA was found in the perfusate and the liver parenchyma during tissue sampling in this study (34). DHOPE also quantified intracellular ATP as a marker of the energy status and viability of the grafts pre- and post-DHOPE (35).

ALT, HMGB-1, and cfDNA were not elevated by extended DHOPE preservation in the perfusate for the livers after re-warming. There were no histological necrotic changes in the DHOPE group. Levels of MDA were not increased in liver biopsies at the end of DHOPE (*p* = 0125) or at the end of reperfusion (*p* = 0.604) when compared to the control static cold storage group. Perfusate levels of HMGB-1, cfDNA, TNF-α, and IL-6 remained low in both livers during the entire period of DHOPE. The reduction in pro-inflammatory cytokines downregulates chemokine and chemoattractant production, which in turn dampens the immune reaction in the DHOPE livers. Von Willebrand factor (vWF), a marker of endothelial injury of the vasculature, was also not increased. vWF, DAMPs and PAMPs are present in endothelial injury and endothelial activation (26). Endothelial activation within inflamed allograft vessels allows the binding of vWF followed by platelets. This then promotes the opening of endothelial junctions and facilitates leucocyte transmigration to the site of liver injury and inflammation, leading to immunothrombosis and potential allograft demise. DAMPs generated are recognized by pathogen recognition receptors (PRRs), such as Toll-like receptors (TLRs) and cytoplasmic Nod-like receptors (NLRs), which drive an immune response.

The immune cells such as mast cells, macrophages, dendritic cells, innate lymphoid cells, and basophils recruited to site of inflammation and injury by chemokines express PRRs on their surface, sensing and binding with PAMPs and DAMPs. PRR activation leads to the production of proinflammatory cytokines including TNF-α, IL-1, vasoactive amines (e.g., histamine and serotonin), nitric oxide (NO), reactive oxygen species (ROS), neuropeptides, and arachidonic acid metabolites (e.g., prostaglandins and leukotrienes). This cascade of pro-inflammatory cytokine production induces damage and inflammation leading to the disruption of the integrity of macro- and micro- endothelial barriers.

NMP and HMP diminish the progression of endothelial damage and generation of DAMPs that drive a pro-inflammatory immune reaction. Tietjen et al. (36) have shown that with the use of *ex vivo* machine perfusion there are windows of opportunity to deliver therapeutics to the organ directly. The group targeted endothelial cells as their primary targets for reducing ischaemic-reperfusion injury (IRI) in renal allografts during NMP. Therapeutics have been developed to target endothelial cell injury and improve long-term outcomes. The group used nanoparticles (NP) to serve as a delivery mechanism of key medications, using surface conjugation of an anti-CD31 antibody (also known as anti-platelet endothelial cell adhesion molecule-1 (PECAM-1) antibody) to enhance targeting of NPs to graft endothelial cells of human kidneys undergoing NMP.

Similar studies have also used NMP to deliver therapeutics to reduce the immune response driven by IRI. Thompson et al. used multi-potent adult progenitor cells (MAPCs) to minimize IRI (37). MAPC-treated kidneys demonstrated improved physiological parameters when compared to the control group. Parameters including improved urine output (*p* = 0.009), reduced expression of novel injury biomarker, Neutrophil gelatinase-associated lipocalin, (NGAL) (*p* = 0.012), and improved microvascular perfusion and down regulation of pro-inflammatory cytokines. Laing et al. utilized NMLP to deliver MAPCs, demonstrating that these MAPCs can be delivered to the liver prior to host immune cell involvement and with maintenance of continuous perfusion. The study showed trans-endothelial migration of MAPCs into vasculature and parenchymal endothelium. Whilst in the endothelium, MAPCs secreted soluble factors that would have anti-inflammatory and immunomodulatory benefits in a human model of liver transplantation (38).

## Normothermic Machine Perfusion

Early NMLP studies (39, 40) describe how perfusing the graft at a physiological temperature has been revived and is at the forefront of “bench to bedside” practice in transplantation. In contrast to Simple cold storage (SCS) is a process by which the preservation solution is infused into the organ and then stored statically at hypothermic temperatures; the principles of NMLP is to maintain physiological conditions by supplementation of key metabolic substrates during perfusion of the organ. This is obtained with inflow of human blood or an oxygen-carrying blood substitute at 37°C, which simulates physiological conditions. In this environment, cell metabolism is maintained thanks to normal oxygen tensions within the perfusate, preventing ischaemic changes and providing the substrate for ATP production. In this setting, feasibility and safety were demonstrated and IRI and its consequences were shown to be reduced (41–43).

The main features and potential appeal of NMLP is to offer the possibility for assessing graft viability, for graft reconditioning and for improving high-risk potential liver allografts (44). This ability has been crucial, as many potentially transplantable livers might otherwise be discarded if deemed suboptimal without the means to recondition and improving the graft function prior to assessing the grafts' function in simulated physiological conditions.

There are four NMLP devices that are available: the OrganOx metra® (OrganOx Ltd., Oxford, UK), the Liver Assist (Organ Assist®, Groningen, the Netherlands), the Cleveland circuit (Cleveland Clinic, Cleveland, Ohio, US) and the OCS™ Liver System (Transmedics, Andover, Massachusetts, US). The mechanism of action is the same with four common main components: an oxygenator, a blood reservoir, a pump that can be single or double for hepatic artery and portal vein inflow and a heat exchanger.

Early human application of NMLP in the UK was documented by the Birmingham Machine Perfusion Group (45–47). The group compared outcomes of NMLP of DBDs and DCDs liver allografts to a matched control SCS group: the first 20 patients were transplanted with NMP primed grafts and described adequate outcomes (48). No grafts in either arm of the trial had primary non-function as a complication. Three patients' grafts (15%), had early allograft dysfunction (EAD) in the NMLP group compared to nine patients (23%) in the control SCS group. Median intensive care unit and hospital stays were comparable between the NMLP and SCS groups. Four DBD-NMLP patients in the trial developed anastomotic biliary strictures, which were treated with stenting. Patient survival at 1 year in the NMLP group was 95%, with 1 death at 9 months accounted for by an alcohol abuse relapse (46).

Bral et al. reported preliminary single-center North American experience using the OrganOx metra® device with nine liver grafts, which were matched with SCS grafts (49). There was no statistically significant difference in peak AST levels in the first 7 days in NMLP and SCS preserved grafts (*p* = 0.52), and there was no statistically difference in bilirubin levels during the first 7 days between the groups (*p* = 0.35). There was also no statistically significant difference in graft survival at 30 days in an intention-to-treat analysis (*p* = 0.25). Intensive care and hospital stays were higher in the NMLP group. This preliminary experience demonstrates feasibility as well as the potential technical risks of NMLP and highlights a need for larger, randomized studies with long-term follow up to assess functionality of NMP in the clinical setting (50).

Nasralla et al. published the result of a large multinational, open-label, two-arm, parallel randomized controlled trial on NMLP versus SCS (50). The trial randomized 334 livers, allocating 164 to SCS and 170 to NMLP using the NMLP OrganOx metra® device. Eventually, 101 SCS and 121 NMLP were successfully transplanted. They reported that peak AST levels during the first 7 days after LT were reduced by 49.4% in the NMLP group compared to SCS when adjusted by center and donor type (geometric mean ratio 0.506, 95% confidence interval 0.388–0.659; *P* < 0.001). There was one case of PNF in the NMLP group, whereas no cases of PNF were reported in the SCS group. EAD rates were available in 216 recipients: the odds of developing EAD in the NMLP arm (12 out of 119) were 74% lower than the SCS arm (29 out of 97; odds ratio 0.263, 95% confidence interval 0.126–0.550; *P* < 0.001). The median bilirubin level in the first week after surgery was lower in NMLP recipients (2.25 mg dl^−1^, 95% confidence interval 1.23–4.28) than in the SCS group (2.87 mg dl^−1^, 95% confidence interval 1.52–5.00; *P* = 0.029). There was no difference in median intensive care unit stay (*P* = 0.339) as well as hospital stay (*P* = 0.926) or the need for renal replacement therapy in the first postoperative week (*P* = 0.621) between the two groups. Graft survival at 1 year in the NMLP group was 0.950 (95% confidence interval 0.893–0.977), compared with the SCS 0.960 (95% confidence interval 0.897–0.985) (*P* = 0.695) (50).

The COPE (Consortium on Organ Preservation in Europe) project (51, 52) demonstrated significant reductions in peak AST and EAD rates in NMLP livers. However, it was stated that a larger trial with a longer follow-up period is mandatory to establish and assess whether there is a major difference in graft or patient survival.

Ghinolfi et al. reported the results of 20 livers from donors 70 years old or older randomized with SCS, thus 10 livers were perfused with NMLP Organ Assist® (53). The results showed no PNF in either group. There were two cases of EAD in NMLP and 1 case in SCS. No differences were observed in either a post-operative transaminitis or bilirubin peak in the first 7 post-operative days. In the NMLP arm of the trial, one graft was lost due to hepatic artery thrombosis, but no other vascular complications were observed in any other trial patients. In the SCS group, one patient died due to sepsis. Furthermore, the researchers performed biopsies and demonstrated that there was histological evidence of reduced IRI, demonstrated by a significant decrease in mitochondrial volume density and intracellular lipid droplets at the end of transplantation in the NMLP group vs. the SCS group (*P* < 0.001). All these reports are summarized in Table 1.

**Table 1:** Outcomes of Normothermic machine perfusion (NMP).

**References**	**Study Type**	**NMP device**	**Donor type**	**N^**°**^ graft NMP vs. SCS**	**Graft Survival at 180/365 days NMP vs. SCS**	**Patient Survival at 180/365 days NMP vs. SCS**	**Median AST peak 7 days**
Ravikumar et al. (46)	Observational	OrganOx metra®	DBD + DCD	20 vs. 39	180 days: 100% vs. 97.5%	180 days: 100% vs. 97.5%	417 vs. 902
Perera et al. (45)	Observational	OrganOx metra®	DCD	1 vs. 0	365 days: 100%	365 days: 100%	NA
Mergental et al. (47)	Observational	OrganOx metra®	DBD+DCD	5 vs. 0	180 days: 100%	NA	NA
Bral et al. (49)	Nonrandomized pilot study	OrganOx metra®	DBD+DCD	10 vs. 30	180 days: 80% vs. 100%	180 days: 89% vs. 100%	1,252 vs. 839
Nasralla et al. (50)	RCT	OrganOx metra®	DBD+DCD	121 vs. 101	365 days: 95% vs. 96%	365 days: 94.9 vs. 95.8%	167.5 vs. 318.5
Ghinolfi et al. (53)	RCT	Liver Assist®	DBD	10 vs. 10	180 days: 90% vs. 90%	180 days: 100% vs. 90%	709 vs. 574

*RCT, randomized controlled trial; DBD, donor brain death; DCD, donor cardiac death; SCS, static cold storage; AST, aspartate aminotransferase*.

## Normothermic Machine Perfusion For Organ Preservation

As the demand for livers for transplantation grows worldwide, maximizing graft utilization is paramount. In this setting, NMLP allows groups to assess the liver viability and to chronologically evaluate the quality of at-risk grafts requiring restoration. Evidence shows that NMLP could play a role in reconditioning and restoring organs such as DCD or steatotic livers to optimize potentially transplantable liver allografts. The latter are considered to be grafts from ECDs, especially with the biopsy-proven macrosteatosis lipid concentration at 30–40%. In fact, macrosteatotic livers have been shown to have the poorest graft outcomes in terms of PNF and graft dysfunction, and consequently many of them are declined. The reason for this poor function seems to be excessive cytoplasmic fatty acids, which enhance lipoperoxidation, thus releasing more free radicals and reactive oxygen species (54). This induces disruption of cells, releasing PAMPs and DAMPs and therefore triggering the activation of TLRs (specifically TLR2 and TLR4), RIG-I-like receptors and NOD-like receptors, which all drive a pro-inflammatory response (55).

In experimental models, NMLP has been associated with reduction of triglyceride content as well as increased bile and urea production, and this process is enhanced with the implementation of liver defatting protocols (56). NMLP alone in human steatotic livers did not demonstrate encouraging results; a Liu et al. study reported that after 24 h of NMLP there was no reduction in tissue steatosis (57, 58). Pharmacological-induced defatting feasibility trials have shown some promise in widening the donor pool. Nagrath et al. reported that after 48 h of NMLP, administering a defatting cocktail produced a 35% reduction in the intracellular lipid content within the livers for this model (59, 60). Other studies of pharmacologically induced defatting agents during NMLP used 10 mmol of L-carnitine added to the perfusate in order to improve mitochondrial oxidation of fatty acids, obtaining a 10% reduction in macrosteatosis (61).

Boteon et al. allocated ten discarded steatotic livers into two NMLP groups: the treatment group had the perfusate supplemented with a defatting cocktail, whereas the control group was without supplementation. The defatting cocktail reduced tissue triglycerides by 38% and macro-vesicular steatosis by 40% over 6 h. Moreover, grafts in the defatting group displayed augmented and improved metabolic functional parameters such as urea production (*P* = 0.03), lower release of alanine aminotransferase (*P* = 0.049), and higher bile production (*P* = 0.008) with a higher bile pH (*P* = 0.03). Furthermore, the treatment reduced expression of key markers of IRI as well as activation of immune cells (CD14- found in neutrophils and macrophages; CD11b- found in NK cells and macrophages) and reduced the release of pro-inflammatory cytokines in the perfusate, particularly TNF-α and IL-1β (62).

NMLP has proven to maximize organ utilization and offer the opportunity for organ reconditioning and rehabilitation by circumventing the mechanisms of IRI and their deleterious consequences to the allograft. Further, the defatting of human grafts opens the possibility of treating declined steatotic livers and enhancing their utilization rate. Defatting trials have shown improvements in hepatic microcirculation, downregulation of Kupffer cells, and reduced release of inflammatory mediators during reperfusion, resulting in a reduction in subsequent reperfusion tissue injury. Although these findings are promising, rigorous research with clear long-term outcome data is required before it can be considered a widely accepted clinical option (63).

## Immune Response and NMLP

NMLP significantly improves post LT outcomes with a reduction of the IRI in CIT. NMLP therefore enhances ATP restoration by decreasing the anaerobic metabolism of cells, leading to less accumulation of metabolites such as lactate, which affect the acid-base balance within the hepatic microenvironment and often enhance proteolytic enzymatic activity and liver tissue degradation (64). Jassem et al. detailed results regarding the immune response after NMLP (65). The study matched 12 DBD grafts perfused with NMLP and 27 DBD grafts preserved with SCS. At the end NMLP or SCS, they performed serial biopsies pre- and post-reperfusion for both histological and transcriptomic analysis of the perfusate and parenchyma from the livers in the study. Intrahepatic lymphocytes were extracted were recognized to be representative of liver-resident lymphocytes (66). The group found that in the SCS group there was a higher level of gene expression of immune-related genes, in particular pro-inflammatory cytokines, and also increased expression of genes involved in humoral immunity, platelet activity and neutrophil chemotaxis, when compared to the NMLP group. Moreover, several genes linked to the stress response and cell apoptosis, such as thrombomodulin (THBD) and IFN-γ, were up-regulated in the SCS cohort but not in the NMLP livers in the post-reperfusion phase of the study. The study assessed the effect of NMLP on the differentiation of T helper cells: Th1 (IL-2, IL-17, IFN-γ), Th2 (IL-4) as well as on Treg (IL-10 and TGF-β) cytokine production by intrahepatic CD4+. CD8+ T cells have also shown to secrete cytokines which effect T cells differentiation. NMLP grafts had significantly lower amounts of CD4+ T cells producing IL-4 (*p* < 0.05), IL-2 (*p* < 0.001), IFN-γ (*p* < 0.05), and IL-17 (*p* < 0.0001). There were no significant differences found in the production of the anti-inflammatory, regulatory cytokines IL-10 or Transforming Growth Factor-Beta (TGF-β). NMLP significantly decreased the proportions of CD8+ T cells subsets producing IFN-γ (*p* < 0.001), while slight reductions in IL-17 were also observed. There were no changes in IL-2 production by CD8+ T cells after NMLP. Studies have identified that within the perfusate and liver tissue samples in both NMP and SCS there is a significant increase in regulatory T helper cells and regulatory cytokines implementing immune tolerance in liver grafts (65, 67). We also see a statistically significant abundance of intrahepatic Tregs in NMP when compared to SCS (4.36% ± 3.27 vs. 1.9% ± 1.8, *p* = 0.0156), and in conjunction with the presence of IL-2 this promotes an expansion and proliferation of the Treg subset. In regards to the clinical parameters of long-term functionality and survival of graft measured in the study, the peak AST and peak INR in the first 7 post-operative days were significantly lower in the NMLP group compared with the SCS group (*p* < 0.01 and *p* = 0.07, respectively), thus suggesting that the NMLP group had grafts with a greater functionality and survival probability than the SCS group (65).

Intrahepatic lymphocyte populations have been identified as being integral in initiating the innate and adaptive immune responses. Certain lymphocytes subsets have a varied prevalence in the NMP and SCS. CD3^−^CD19^+^ B cells, CD3^+^CD4^+^ T cells and CD3^+^CD56^+^ Natural Killer (NK) T (NKT)-like cells were statistically similar in the liver tissue and perfusate of both populations in both NMP and SCS (68). Intrahepatic NK cells are contributors in acute and chronic allograft rejection and are a key cell subset in amplifying the inflammatory process. NK cells were more prevalent in tissue and perfusate from the SCS samples when compared to NMP samples. NMP livers showed a significant up-regulation of genes involved in immune-trafficking compared to SCS in both pre-reperfusion and post-reperfusion stages. In biopsies collected pre- and post-reperfusion, the Jassem et al. study demonstrated significantly lower quantities of apoptotic and necrotic cells in NMLP compared to SCS, as well as reduced quantities of neutrophil clusters within the parenchyma when NMLP livers were compared with SCS livers in both pre- and post-reperfusion stages. The group concluded that NMLP minimizes inflammation and cell death, and promotes liver restoration and regeneration (65).

## The Future of Organ Preservation

Sub-normothermic machine liver perfusion (SNMLP) has become a prominent technique in preserving livers at lower temperatures on the perfusion circuit. A research group stored the human liver graft at −4°C with the supercooling method followed by sub-normothermic machine perfusion. This technique could extend up to 27 h of *ex vivo* preservation of the liver, without altering the viability of the organ. In super-cooling livers, the parenchyma can withstand the stress of simulated transplantation by *ex vivo* normothermic reperfusion with blood (69). The results have shown some promising clinical outcomes (70, 71).

Other studies like Eshmuminov et al. have attempted to increase the length of time for reconditioning on NMP and have demonstrated graft viability on animal models perfused for 7 days without the need for additional blood products or perfusate exchange (72). This novel approach was conducted using ten human livers declined for transplantation. After 7 days of perfusion, 6 out of 10 human livers showed preserved function as indicated by bile production, synthesis of coagulation factors, maintained cellular energy (ATP) and an intact liver structure (72).

The boundaries of NMLP have attempted to be pushed to meet the demands of transplantation as well as improving outcomes of these marginal livers used. The proof of concept study developed by Wu et al. raises the possibility of an ischaemia-free organ transplantation (IFOT). In essence, by removing the total element of IRI by preventing it whilst continually maintaining the blood supply for the organ at a physiological temperature throughout the procurement period until implantation, we may be able to eliminate the threat of graft dysfunction secondary to IRI. The group maintained NMLP during procurement and preservation until implantation and the recipient experienced no post-reperfusion syndrome. Initial investigations including liver function tests as well as histological analysis demonstrated a low injury to hepatocytes and vascular and biliary epithelium during preservation and post-transplantation. Moreover, they stated that the inflammatory cytokine levels were much lower in IFOT than those in conventional procedures (73).

Undoubtedly, there is an impending influx of innovative technologies will open new horizons for organ preservation and restoration and in return reduce mortality on transplant waiting lists. Despite clinical trials in the field being in their infancy, the results so far have been very encouraging with promising graft and patient outcomes. However, with few medical governing bodies embracing and introducing machine perfusion preservation for transplantable organs into their guidelines, further work in the form of multi-center randomized controlled trials with a focus on long-term outcome data is required in order to establish best clinical practice for liver preservation and restoration.

## Author Contributions

AP and D-CO-B conducted the literature research and co-wrote the paper. VR was aided in the writing of the immunological aspects of the paper as well as performed integral editing of parts of the review. MP and DM supervised and edited the overall review up to submission. All authors contributed to the article and approved the submitted version.

## Conflict of Interest

The authors declare that the research was conducted in the absence of any commercial or financial relationships that could be construed as a potential conflict of interest.

## Abbreviations

CIT: Cold Ischemia Time
DAMPs: Damage Associated Molecular Patterns
DBD: Donation after Brain Death
DCD: Donation after Cardiac Death
DHOPE: Dual Hypothermic Oxygenated Perfusion
EAD: Early Allograft Dysfunction
ECD: Extended Criteria Donors
HMP-: Hypothermic Machine Perfusion
HOPE-: Hypothermic Oxygenated Perfusion
IFN: Interferon
IFOT: Ischaemia Free Organ Transplantation
IL: Interleukin
IRI: Ischaemic Reperfusion Injury
LT: Liver Transplantation
MAPCs: Multipotent Adult Progenitor Cells
MP: Machine Perfusion
NLR: Nucleotide-binding Oligomerization Domain (NOD)-like receptors
NMLP: Normothermic Machine Liver Perfusion
PAMPS: Pathogen-Associated Molecular Patterns
PNF: Primary Non-Function
PRR: Pathogen Recognition Receptor
RIG-I: Retinoic acid-Inducible Gene-I-like receptors
ROS: Reactive Oxygen Species
SNMP: Subnormothermic Machine Perfusion
TLR: Toll Like Receptors
TNF: Tumor Necrosis Factor
WIT: Warm Ischaemia Time.
